# Introducing social determinants of health to the Alzheimer's Disease Research Center network: Development and implementation in the Uniform Data Set

**DOI:** 10.1002/alz.70279

**Published:** 2025-05-23

**Authors:** Megan Zuelsdorff, Erin L. Abner, Joyce E. Balls‐Berry, Gregory A. Jicha, Serggio Lanata, Gladys E. Maestre, Monica Rosselli, Shana D. Stites, Rachel A. Whitmer, Consuelo H. Wilkins, Lisa L. Barnes

**Affiliations:** ^1^ School of Nursing University of Wisconsin‐Madison Madison Wisconsin USA; ^2^ Wisconsin Alzheimer's Disease Research Center University of Wisconsin School of Medicine and Public Health Madison Wisconsin USA; ^3^ Department of Epidemiology and Environmental Health University of Kentucky Lexington Kentucky USA; ^4^ Sanders‐Brown Center on Aging University of Kentucky Lexington Kentucky USA; ^5^ Department of Neurology Washington University School of Medicine St. Louis Missouri USA; ^6^ Knight Alzheimer's Disease Research Center Washington University School of Medicine St. Louis Missouri USA; ^7^ Departments of Neurology Behavioral Science, and Neuroscience University of Kentucky Lexington Kentucky USA; ^8^ Department of Neurology University of California San Francisco California USA; ^9^ Memory and Aging Center University of California San Francisco California USA; ^10^ Department of Neuroscience University of Texas Rio Grande Valley School of Medicine Harlingen Texas USA; ^11^ South Texas Alzheimer's Disease Research Center University of Texas Rio Grande Valley School of Medicine One West University Boulevard Brownsville Texas USA; ^12^ Department of Psychology Florida Atlantic University Davie Florida USA; ^13^ 1Florida Alzheimer's Disease Research Center Florida Atlantic University Gainesville Florida USA; ^14^ Department of Psychiatry University of Pennsylvania Philadelphia Pennsylvania USA; ^15^ University of Pennsylvania Alzheimer's Disease Research Center University of Pennsylvania Philadelphia Pennsylvania USA; ^16^ Department of Public Health Sciences University of California Davis California USA; ^17^ Alzheimer's Disease Research Center University of California Sacramento California USA; ^18^ Office of Health Equity Vanderbilt University Medical Center Nashville Tennessee USA; ^19^ Division of Geriatric Medicine Department of Medicine Vanderbilt University Medical Center Nashville Tennessee USA; ^20^ Vanderbilt Alzheimer's Disease Research Center Vanderbilt University Medical Center Nashville Tennessee USA; ^21^ Departments of Neurological Sciences and Psychiatry and Behavioral Science Rush University Medical Center Chicago Illinois USA; ^22^ Rush Alzheimer's Disease Center Rush University Medical Center Chicago Illinois USA

**Keywords:** cognitive aging, dementia, health disparities, social drivers of health

## Abstract

The Alzheimer's Disease Research Centers (ADRCs) consortium represents a critical locus of research on Alzheimer's disease and related disorders (ADRD) prevention, diagnosis, and intervention. Through the National Alzheimer's Coordinating Center's (NACC) standardized protocol, the Uniform Data Set (UDS), ADRCs have collected rich, harmonizable clinical and cognitive data. However, the collection of social data has been sparse and Center specific, constraining ADRD science that addresses research priorities on social determinants of health (SDOH) and health equity. Capitalizing on the transition to a revised UDS version 4, an interdisciplinary committee representing 10 ADRCs reviewed the literature and instrumentation, ultimately creating a brief module covering multiple domains and levels of exposure required for mechanistic studies of SDOH and brain health. This article offers rationale, empirical support, and guidance for using the selected constructs: transportation security, financial security, social connectedness, health care experiences, and discrimination, as well as recommendations for next steps that each ADRC can take to maximize local and field‐level progress.

**Highlights:**

- Social determinants of health (SDOH) play a role in Alzheimer's disease and related dementias (ADRD) risk, diagnosis, care, and research participation.
- A new module adds SDOH to a revised Uniform Data Set (UDS) for the Alzheimer's Disease Research Center (ADRC) consortium.
- UDS SDOH include transportation, socioeconomic status, social relationships, health care, and discrimination.
- We provide evidence for causal SDOH associations with ADRD and guidelines for use.
- We include recommendations for next steps and expanding the impact of the SDOH module.

## 1 OVERVIEW

Social determinants of health (SDOH) are broadly defined as the conditions in which people are born, grow, live, work, and age,^1^, ^2^ and decades of sociological and public health research demonstrate the critical role of these determinants in health and disease.^3^, ^4^ SDOH are generally conceptualized as factors that are beyond individual control, shaped by a complex and linked system of political, environmental, and social forces; they are sometimes termed social *drivers* of health to emphasize their modifiability through structural and community‐level intervention and resourcing.^3^, ^5^, ^6^ SDOH have recently gained significant attention in the field of cognitive aging,^7^, ^8^ although new momentum is supported by years of evidence that markers of social status, including early‐life education experiences and later‐life wealth, are robust predictors of risk of Alzheimer's disease and related dementias (ADRD).^9^, ^10^, ^11^, ^12^, ^13^ In some cases, such as air pollution exposure or educational opportunity and attainment, SDOH may affect brain health and ADRD risk directly.^14^, ^15^ SDOH also strongly influence an individual's exposure to behavioral and biomedical factors; conceptually, they are “causes of the causes” among middle‐aged and older populations at risk for ADRD.^3^ Through both direct and indirect impacts, SDOH represent a nexus of risk and protective factors foundational to ADRD prevention and intervention across all populations.

That said, SDOH are also critical to understanding and addressing ADRD inequities. Distributions of specific SDOH are frequently systematized by intersections of upstream determinants such as racialization, geography, and social status, referred to as “fundamental causes” or “social determinants of equity” in well‐established public health frameworks.^16^, ^17^ Disproportionate exposure to unhealthy living conditions across SDOH domains and across the life course contributes to health disparities—defined as avoidable and unjust differentials in risk and burden^3^–that impact populations minoritized due to race, sexual orientation, and/or gender identity, as well as rural and socioeconomically disadvantaged communities.^18^, ^19^, ^20^

The National Institutes of Health (NIH)–funded Alzheimer's Disease Research Center (ADRC) consortium collects rich biomedical and behavioral data; since 2005, ADRCs have utilized a standardized data collection protocol known as the Uniform Data Set (UDS). The fourth update of the UDSv4, implemented in 2025, offered a unique opportunity for the consortium's Clinical Task Force (CTF) and the National Alzheimer's Coordinating Center (NACC)^21^ to broaden the scope of clinical information within the UDS^22^ and include key social determinants important for brain health in a new SDOH module. Although brief, the new module's constructs span multiple domains of distal and proximal social factors that, when analyzed in conjunction with downstream behavioral and biomedical data, can illuminate mechanistic pathways to brain health outcomes and disparities.^23^, ^24^ In this article, we describe the process of module development; provide empirical support for the selected constructs as key determinants of dementia risk, diagnosis, care, and research participation; and offer guidance for using the new measures.

## 2 BACKGROUND

In February 2020, a new CTF was established to revise the UDS version 3 (UDSv3). The CTF began by conducting a consortium‐wide survey designed to gather information on what data individual centers were collecting beyond the UDS. Both survey results and information from prior CTFs emphasized an absence of SDOH data as a critical gap in data collection. Although aspects of SDOH were being collected by some centers in the network, and a few teams had innovatively triangulated UDSv3 demographic data to assess participant information such as same‐sex partnership,^25^, ^26^ it was clear that many additional data elements were needed to facilitate research efforts on brain health and equity on a larger scale.

Thus, a SDOH workgroup was established and charged with designing a module that would capture information on the lived experience of participants while limiting the burden on participants and staff. Dr. Lisa Barnes from the Rush Alzheimer's Disease Center and a member of the CTF, was asked to lead the group. She invited Dr. Megan Zuelsdorff from the University of Wisconsin ADRC to co‐chair, and together they invited 11 experts from across the country, representing funded P30 and P20 ADRCs, with knowledge in social determinants, health equity, and methodological approaches to studying health disparities. Committee members included the authors and two ex‐officio members from National Institute on Aging (NIA). The committee met from November 2021 until March 2022 to create the optimal battery and provide recommendations to the CTF and NACC on which, and how, SDOH should be measured. Continuing input from CTF, NACC, and ADRC teams informed the module as its items and wording were finalized. The final SDOH module and other UDSv4 forms are available, along with detailed guidance on administration and data dictionaries, on the NACC website: https://docs.naccdata.org/edc/downloads/udsv4.

### 2.1 Guiding principles for selecting constructs and measures

The UDSv4 adds several new domains including mild behavioral impairment, subjective cognitive decline, and ADRD disease‐modifying treatments, as well as new demographic questions on gender identity, sexual orientation, and multi‐ethnic/racial identities.^27^, ^28^ Brevity of the SDOH module was required to balance new data priorities with minimizing participant and Center staff burden. The committee identified a maximum module time‐to‐completion of ≈20 min. That time constraint informed the number of constructs selected, and the number of survey items included within each construct. Prior to the selection of any specific constructs or associated operationalization, the committee identified several foundational principles for selection based on member consensus and CTF/NACC guidelines.

#### 2.1.1 Selection criteria for SDOH constructs and their measurement

First, selected constructs draw from multiple domains of SDOH as they are represented by existing frameworks, most notably the World Health Organization's Healthy People 2030 model^1^ and the NIA's Health Disparities Framework.^23^ Although exposure to socioenvironmental risk and protective factors is frequently systematized at a population level, individual experiences are unique. Collecting data across multiple domains allows for the assessment of a range of exposure profiles and intersections between factors. There was also the need to consider protective factors not inherently correlated with socioeconomic advantage, such as social connectedness.^29^, ^30^, ^31^

Second, construct selection prioritized strong and plausibly causal empirical associations with ADRD‐related outcomes. In addition to SDOH showing direct associations with brain health, dementia, or cognitive impairment, the committee also considered social–environmental conditions that influence major ADRD risk factors and/or access to ADRD research, diagnosis, and care. Social conditions that shape these latter outcomes may have crucial implications for equitable assessment and effectiveness of intervention at all stages of ADRD prevention and treatment.^32^

Third, construct selection was tailored to the current nationwide NACC cohort. ADRCs are devoting substantial resources to removing institutional barriers to the recruitment and retention of participants, and these efforts are yielding an ADRC‐wide cohort that is increasingly diverse. Generally, however, the committee prioritized constructs likely to already be well distributed across ADRC cohorts, ensuring that these new exposure variables would comprise sufficient variation to analyze in the near term. That said, there was consensus that experiences of discrimination must be included given robust theoretical and empirical support for discrimination as a race‐based stressor and determinant of brain aging, particularly within the minoritized communities that experience disproportionate ADRD risk and burden.^33^, ^34^, ^35^

Fourth, the selection process prioritized constructs for which validated instrumentation was already available, and/or which are already measured in another large cohort study of aging. It was unfortunately not possible to include the full‐validated instrument for any construct given time and length constraints for the SDOH module as a whole, and subsets of questions from those instruments included in the module do not themselves constitute validated measures. However, utilizing items and domains from validated and/or in‐use measures should facilitate comparability and harmonizability with participants and data from other cohorts.

Finally, the committee prioritized measures of lived experiences that cannot be readily accessed or measured by linking ADRC participant research data to public datasets. Through innovations and expertise in geocoding, many ADRD investigators have used addresses and residential histories to establish associations between ADRD outcomes and life course area‐level SDOH including ethnoracial segregation,^36^ school quality,^37^ environmental contaminants,^38^ neighborhood disadvantage,^39^ access to green spaces,^40^ and proximity of social activity hubs.^41^ Additional high‐priority exposures, including neighborhood walkability, nutritious food access, and safety^42^ can also be assessed via data linkage. Using existing geospatial data minimizes questionnaire burden for exposure assessment, leaving room in UDSv4 for complementary questionnaire data on individual‐level experiences likely to mediate or intersect with effects of area‐level exposures.

### 2.2 Empirical foundations and utilization guidelines for selected measures

#### 2.2.1 Transportation security

Reliable access to transportation was identified as a foundational SDOH for older adults and prioritized as a key variable in the new module. Transportation may be most closely tied with the built environment, but is also inextricably linked to health care and social environments. Transportation security is likely to be highly salient for ADRC participants: although participation in an ADRC cohort or other intensive cognitive aging study may self‐select for individuals with reliable means of transportation at the time of enrollment, these means can change with advancing age. Common late‐life events, such as the onset of functional limitations or the loss of a ride‐providing spouse, sibling, or friend, can influence transportation access.^43^ These life events impact older adults across sociodemographic strata, with nearly 20% of adults over age 65 experiencing transportation insecurity; however, evidence suggests that minoritization and socioeconomic disadvantage associate with a greater likelihood of transportation precarity.^44^ This is a crucial consideration for both public health and ADRD intervention. The new SDOH module will facilitate within‐Center and NACC‐level investigation of transportation security as an upstream determinant of cognitive health and research participation in aging communities, and an intervenable moderator of geographic disparities in ADRD outcomes including those experienced by rural communities.^45^ Transportation insecurity has rarely been examined as a risk factor for cognitive impairment or dementia, particularly in longitudinal analyses necessary to parse directionality, but in at least one large population‐based study, driving cessation predicted faster cognitive decline over the subsequent 10‐year period.^46^ A causal relationship between transportation insecurity and cognitive impairment is plausible given that transportation access empirically associates with several modifiable behavioral and biomedical ADRD risk factors. Most notably, a number of studies report that transportation insecurity strongly predicts social isolation^47^, ^48^ and nearly doubles the risk of depressive symptoms^49^ in older populations. Transportation insecurity may also impede recruitment and retention for ADRD studies relying on in‐person clinic visits; lower transportation mobility is associated with reduced research participation and willingness to join clinical studies and trials.^50^

To capture transportation security and changes therein, the SDOH module includes five questions on individual‐level transportation conditions and experiences. Two questions focus on general access and require a binary yes/no response: *Do you or someone in your household currently own a car*, and *Do you have consistent access to transportation*. Car ownership and overall mobility are distinct and may independently predict health and well‐being outcomes given the importance of ride‐sharing and non‐kin transportation support,^48^ and these relationships may vary by sociodemographic factors such as gender, race/ethnicity, socioeconomic status, and rural/urban residence. Given the dearth of research on transportation access and cognition, we recommend exploring distinct predictive capabilities of each item. The remaining three questions are drawn from the Transportation Security Index^51^ and capture recent (past 30 days) transportation‐related experiences that may mediate impacts of transportation access on health and health behaviors in aging populations: *How often were you not able to leave the house when you wanted to because of a problem with transportation*, *How often did you worry about whether or not you would be able to get somewhere because of a problem with transportation*, and *How often has a lack of transportation kept you from medical appointments or from doing things needed for daily living?* Likert‐scale responses on frequency of negative experiences can be summed for these latter three questions, to create an overall index where higher scores indicate greater transportation security.

#### 2.2.2 Financial security

Social gradients of health, wherein socioeconomic markers such as formal educational attainment, occupational status, and wealth robustly predict diverse health outcomes across the life course, are well established. Innovative approaches to studying early‐life educational experiences as a source of cognitive resilience and a population‐level social determinant of dementia disparities have harnessed self‐reported attainment data, cognitive tests of literacy, and geocoded dimensions of historical school conditions to assess educational experiences within cognitive aging cohorts.^11^, ^52^, ^53^ Other socioeconomic markers are underassessed in ADRC‐based studies, but have been studied in relation to cognitive ability and dementia risk in many large, population‐based cohorts,^12^, ^54^, ^55^ ^,^ and interdisciplinary evidence supports causal mechanisms that should be prioritized for analysis within the expanded NACC dataset. For example, economic advantage is inextricably linked with SDOH such as safe neighborhoods, health care, and access to nutritious foods, green spaces, and leisure time required for health behaviors and individual‐level lifestyle changes to reduce ADRD risk.^56^ Financial resources also provide a partial buffer for health crises, onset of functional limitations, and loss of loved ones in later life, allowing individuals to preserve independence and well‐being even in a context of adverse events.^57^ Financial hardship not only limits opportunities for brain‐healthy behaviors, it also increases the likelihood of exposure to chronic stress and correlated physiological detriment: inflammation, vascular disease, and accelerated biological aging.^58^, ^59^ Unhealthy coping behaviors may moderate as well as mediate relationships between financial stress and cognition: Recent findings indicate that negative associations between financial worries and cognitive ability in older adults are exacerbated by social withdrawal and smoking.^60^, ^61^

A diversity of measures have been used to operationalize the overall construct of financial security. Markers of neighborhood‐level socioeconomic disadvantage such as the Area Deprivation Index (ADI),^62^ which link participant addresses to Census data, have grown in prominence within cognitive aging research and broadly associate with cognitive outcomes and neuropathology.^63^ Neighborhood‐level measures are distinct from individual‐level socioeconomic status, which can be measured objectively or subjectively.^64^, ^65^ Among aging research protocols utilizing self‐reported data, most have examined income relative to federal poverty thresholds^54^ or calculated overall wealth/assets.^12^ “Objective” measures, such as quantified income and/or wealth, are arguably less biased by individual perceptions than subjective measures of financial health and adequacy of resources. They may still be prone to measurement error and less able to capture variability in financial adequacy across different regions or family structures. Furthermore, even measures of perceived financial adequacy can miss crucial, context‐dependent sources of social and cultural capital.

The financial security component of the UDSv4 aims to capture several dimensions of economic well‐being and social status. Notably, questionnaire data complement the ADI,^62^ calculated as part of UDSv4 based on residential address and block‐level census data. Within the SDOH module, participants are first asked to provide self‐reported household income from all sources including retirement benefits and financial help from relatives, using categorical response options that span ranges from $0–14,999 to the top bracket, $75,000 and over. Research teams who use these data are advised that it is challenging to combine income data across regions; as an exposure, income data may be most useful at the local ADRC level, where regional cost‐of‐living may be relatively constant across most participants.

Following the income item, five items on current and/or previous financial satisfaction (e.g., How satisfied are you with your current financial situation?) and hardship (e.g., In the last 12 months, have you ended up taking less medication than was prescribed for you because of the cost?) are drawn from the Health and Retirement Study (HRS).^66^ Two of these questions, on difficulties with affording medication and affording food, are also adapted as unique items that cover lifelong financial hardship exposure (e.g., At any time, have you ended up taking less medication than was prescribed for you because of the cost?). The HRS is a population‐based, nationally‐representative study that has supplied data for hundreds of publications on the health of older adults. A multitude of approaches to analyzing financial security items, including as unique predictors and cumulative indices, are well described in studies of diverse ADRD‐relevant outcomes including cognitive function.^67^, ^68^, ^69^ Investigators should review and select a specific approach to quantifying subjective financial satisfaction and/or strain through a priori review of HRS‐based literature relevant to their research question.

Next is a measure of social status: the MacArthur Scale of Subjective Social Status. This well‐validated measure of perceived social and community value robustly predicts mental and physical health outcomes^70^ and is straightforward in its scoring, with rungs of the social “ladder” representing a continuous scale of status. That said, it is notable that subjective social status is empirically and analytically distinct from economic status^70^ and that the magnitude of effect on health outcomes including ADRD‐related outcomes may be population specific.^71^

Mother's educational attainment is included as an established indicator of familial social status and early‐life socioeconomic conditions. Modeled as a categorical variable, it is a powerful predictor of diverse health outcomes including cognitive function and dementia risk.^72^

#### 2.2.3 Social connectedness

Social isolation has received increasing attention as a modifiable risk factor for cognitive aging, empirically associating with cognitive test performance, rates of decline, brain aging, and dementia risk.^29^, ^30^, ^31^ With a sensitive period located in later life,^6^ social connectedness offers exciting potential for translation into non‐pharmacological interventions for ADRC participants and their peers. The wealth of biomedical and biomarker data already collected by many Centers will facilitate the identification of key mechanistic pathways. Defined broadly, social connectedness has been examined as a direct salutogenic exposure, and as a moderator that reduces cognitive risk related to social and psychological stress^73^, ^74^ and other adverse exposures including pathology burden^75^ and diabetes.^76^ Social networks shape at least two distinct facets of connectedness likely to explain cognitive benefits. First, social relationships can provide reliable access to key emotional or material investments that support health behaviors and reduce sequelae of life stressors.^73^ Conversely, the perceived unavailability of companions can present as a stressor: loneliness predicts not only poorer cognitive health but also biomedical ADRD risk factors including inflammation and depressive symptoms.^77^ Second, social engagement (participation in interactive social situations) can provide environmental enrichment through cognitive stimulation—plausibly generating more robust and resilient neuronal connections and providing cognitive reserve for participants with preclinical AD or other signs of brain aging. Empirical evidence for social embeddedness as a predictor of preserved cognition in the presence of neuropathology spans nearly two decades^75^ but a dearth of datasets that include both social and neuropathological data have constrained replication across cohorts. In the interest of nuanced mechanistic research that can parse distinct exposures and pathways of influence to better inform clinical‐ and policy‐level intervention, the new SDOH module measures three constructs of connectedness that are relevant to cross‐disciplinary research and translation. This includes self‐reported data on individual feelings of loneliness, social interactions across life spheres, and neighborhood and community characteristics that facilitate or hamper social relationships and activities.

The first five questions, drawn from the DeJong Giervald Loneliness Scale,^78^ have been used as an index to explore loneliness as a predictor of cognitive outcomes including rate of decline and risk for dementia, and mediation of these pathways by depressive symptoms and hippocampal volumes.^79^, ^80^ Although loneliness is a subjective internal process and not a classical social determinant of health, loneliness items were included as a complement to social activity because level of isolation may be hard to fully assess with traditional measures of network size and engagement: even as networks shrink during later life, key benefits of connection such as social support (both material and emotional) may remain salient.^81^

The next four questions address social activity across four domains: participants are asked about the frequency of engagement with parents, children, close friends, and other groups/activities (e.g., religious activities, educational activities, volunteer work). These items have been included in the visit protocols for diverse Chicago‐based cohorts including the Rush ADRC's African American Clinical Core, and associate with cognitive decline, risk for mild cognitive impairment (MCI) and dementia, and cognitive reserve.^31^, ^75^, ^82^ The items are constructed for calculation of an index score of engagement frequency. However, there is evidence suggesting that cognitive benefits are not equal across all domains of engagement (e.g., contact with friends is associated more positively with cognition than contact with family members),^83^ and relationships between social activities and health may be non‐linear.^84^, ^85^

The final two questions address participants’ perceived safety in their home and in their neighborhood. Community safety has received increasing attention as an important social determinant of well‐being for older adults.^86^ In addition, perceptions of neighborhood safety and cohesion associate with cognitive function and decline over time,^87^, ^88^ with hypothesized pathways through changes in social engagement and other brain‐healthy behaviors such as physical activity.^89^ Perceived lack of safety within one's own home, meanwhile, associates with additional risk factors including psychosocial stress and poor mental health.^90^ Responses for home and community safety items can be used in tandem or individually. If safety concerns are discovered, local institutional review board protocols or center‐specific standard operating procedures for solutions to mitigate harm should be utilized. In some cases it may also be helpful to allocate resources to collaborate with social workers or community health workers who can make referrals to agencies that provide in‐home assistance or support.^91^

#### 2.2.4 Experiences with the healthcare system

A recent study out of the nationally representative, population‐based HRS cohort found that more negative experiences with health care predicted increased risk of dementia across a 12‐year follow‐up period.^92^ Although few studies have examined such relationships explicitly, the rationale is strong. An updated systematic review of modifiable risk factors finds that 14 risk factors across the life course account for nearly half of all dementia cases globally.^6^ The review situates these largely downstream behavioral and biomedical factors firmly within social and structural contexts, placing explicit emphasis on the crucial role for health care access in the modifiability of risk and protective factors by individuals. Among a list of specific actions recommended for public health and clinical practitioners to reduce risk and burden, a majority target health care policies and practices rather than individual behaviors: making hearing aids accessible, treating depression and vision loss, providing smoking cessation guidance and tools, and detecting and managing hypertension and hypercholesterolemia.^34^ The message is clear—individuals cannot address these conditions without support from health care systems and reliable access to therapies, medications, and sensory aids.

It is important to note that insurance coverage does not wholly predict reliable or timely health care access for these needs. Between 10% and 15% of Medicare enrollees report difficulties scheduling appointments and filling prescriptions.^93^ Data from the Kaiser Family Foundation indicate that 20% of insured adults have postponed or skipped medical care such as hearing aid maintenance and mental health therapies due to cost alone.^94^ Furthermore, some populations are disproportionately likely to report challenges including caregivers, minoritized communities, socioeconomically disadvantaged individuals, and rural residents.^93^ ADRC‐based research and clinical teams are frequently tasked with providing “lifestyle” recommendations for ADRD prevention to both individuals and local communities, but must do so without any data on the status of their participants’ health care access and/or needs. At the Center level, addressing this gap in knowledge can help investigators and staff provide post‐visit feedback and recommendations tailored to the needs of their participants, strengthen institution‐community relationships and trust, and ultimately expand the usefulness of ADRCs as a community resource. At the field level, understanding the barriers middle‐aged and older adults face as they attempt to follow guidance for healthy bodies and brains will be critical to designing policy‐ and clinical‐level interventions that narrow, not widen, social disparities.

As such, the new SDOH module includes five items representing key indicators of health care access. Participants are asked four specific questions on past‐year frequency of occurrence: *how often did you delay seeking medical attention for a problem that was bothering you*, *how often did you experience challenges in filling a prescription*, *how often did you miss a follow‐up medical appointment that was scheduled*, and *how often did you follow a doctor's advice or treatment plan when it was given*. Responses for this item can be summed to create an index of health care accessibility. A fifth question asks about general perceptions of access: *Overall, which of these describes your health insurance, access to health care services, and access to medications?* The possible responses focus on the level of adequacy, ranging from “Not available to any extent” to “Exceeds my needs.”

#### 2.2.5 Discrimination

Self‐reported experience of discrimination is a novel and understudied sociocultural variable in cognitive aging research. Although it has been linked to a number of poor physical and mental health outcomes throughout the lifespan,^95^ it is only recently emerging as an important influence in cognitive aging studies. A majority of studies have documented that experiences of discrimination are associated with worse cognitive performance, cognitive decline, or subjective cognitive decline,^33^, ^34^, ^96^ although some studies have not found an association^97^ or reported an unexpected association with better cognition.^98^ Others have examined potential moderators and mediators, finding that protective factors such as purpose in life and social support moderate the association between discrimination and cognitive function,^96^, ^99^ or that sense of control and perceived constraints mediate the association.^100^ Although this is relatively understudied, proposed mechanisms include inflammation,^101^ allostatic load,^102^ and chronic stress.^103^ A smaller body of literature has begun to examine other brain health outcomes, including plasma biomarkers^104^ and neuroimaging.^105^, ^106^ It is clear that discrimination is an important determinant of health; however, the evidence for the connection with brain health is in its infancy and mixed due to differences in methodology (e.g., cross‐sectional vs longitudinal, discrimination scales vs single questions), age ranges of the population, and the covariates considered.

We included five items from the Everyday Discrimination Scale^107^ (EDS), which have been linked with poor health outcomes including brain health.^34^, ^101^, ^105^ The questions assess mild forms of mistreatment and microaggressions that occur daily, and asks about the axes of identity or characteristics (e.g., race, gender, age, weight, education level) being targeted for discrimination. Information on scoring and utilization in the United States and globally is available.^108^

Two additional questions accompany the EDS items. First, to gather new information on potential determinants of brain health, and given the robust literature on this topic with other chronic health outcomes in minoritized populations,^109^ we added a question on experiences of discrimination in medical encounters: *How frequently do you receive poorer service or treatment from doctors or in hospitals compared to other people?* Responses are based on frequency, ranging from “None or almost none of the time” to “All of the time.” Previous work has found that reports of discrimination in medical settings are strongly correlated with overall societal discrimination,^110^ but given the potentially unique health sequelae (such as medical treatment nonadherence) we recommend examining this item independently of the EDS items.

We also included a question on perceived stressfulness of discrimination experiences: When you have had day‐to‐day experiences like those (listed above), would you say they have been very stressful, moderately stressful, or not stressful? Given the mixed findings on valence of relationships between discrimination and cognitive outcomes,^34^, ^98^ more work is needed on buffering or exacerbation of such effects through examining moderators of discrimination‐health relationships.

### 2.3 Future directions for ADRC investigators and teams

In closing, we highlight crucial steps that centers can take to continue and expand on research directions made possible through the new SDOH module. Above all, the committee wishes to reiterate that this module is not comprehensive and does not cover all social conditions relevant to the health of any, much less all, populations. The new SDOH module is intended as a jumping‐off point for increasingly rigorous and impactful research on the socially‐rooted determinants of ADRD prevention, diagnosis, care, and recruitment science. To that end, we make several recommendations for future research.

First, collection of the module's data is only mandatory at one visit. However, collecting these data at multiple visits will allow for modeling of these data as time‐varying exposures during a period of life when transitions are common: retirement, empty nests, grandparenting, caregiving, loss of loved ones, and illness and recovery. Longitudinal data that capture changes in conditions over time and their attendant impacts on ADRD outcomes will offer important insights for translation to intervention.

Second, centers should be aiming to collect data that represent the lived experiences of the communities they are serving. A key step toward that representation is going to be the addition of population‐salient social determinants instrumentation or even full modules. For example, the new SDOH module is designed for completion by individuals and cannot be completed by a proxy, thereby limiting participation to those participants with relatively intact cognition. Within centers that are enrolling substantial numbers of individuals with advanced MCI and dementia, assessing and understanding social conditions that optimize or jeopardize symptom management and other measures of well‐being may be an invaluable next step. In any case, the utility of incorporating participant and community expertise and feedback into such data expansions cannot be understated.^111^ Much of the importance of this brief SDOH module may lie in its provision of preliminary data toward that kind of tailored protocol development and the research questions it can answer.

Third, it is important that the module is collected for all participants in an ADRC—not only the participants who are traditionally underrepresented in research. Disparities in brain health are well established, and measuring social determinants across race, ethnicity, sexual orientation, and other social identities will foster a deeper understanding of inequities in poor brain health, allowing the field to move beyond explanations of biology as a reason for disparate brain health outcomes, and ultimately suggest targets for redressing inequities. This will be particularly important as the potential outcomes expand beyond cognition and clinical diagnoses to include other important brain health indicators including fluid biomarkers, neuroimaging metrics, and neuropathologic data.

Finally, we recommend that centers look for avenues and opportunities to collect further SDOH data from local community members as a point of comparison for those they successfully enroll as participants. The ADRC cohorts’ potential for selection bias, and relative lack of representativeness and generalizability, is acknowledged as a substantial limitation of existing work.^112^, ^113^ Reweighting participants’ data to local population metrics can offer key insights into impacts of selection bias on SDOH associations with cognitive outcomes; where possible, collecting SDOH data at initial screening or from pre‐enrollment registries will further expand our recruitment science to gain a much greater understanding of who is *not* present in ADRC cohorts. There are populations whose experiences and outcomes are completely absent from the science and potentially from its benefits; social determinants data are likely to offer many useful insights into institutional supports that can be developed to make research and representation more accessible to those who have been historically excluded.

## CONFLICT OF INTEREST STATEMENT

The authors have no conflicts of interest to disclose. Author disclosures are available in the supporting information.

## CONSENT STATEMENT

No consent was necessary.

## Supporting information



Supporting Information
